# Human Cardiac Mesenchymal Stromal Cells From Right and Left Ventricles Display Differences in Number, Function, and Transcriptomic Profile

**DOI:** 10.3389/fphys.2020.00604

**Published:** 2020-06-24

**Authors:** Ilaria Stadiotti, Luca Piacentini, Chiara Vavassori, Mattia Chiesa, Alessandro Scopece, Anna Guarino, Barbara Micheli, Gianluca Polvani, Gualtiero Ivanoe Colombo, Giulio Pompilio, Elena Sommariva

**Affiliations:** ^1^Unit of Vascular Biology and Regenerative Medicine, Centro Cardiologico Monzino IRCCS, Milan, Italy; ^2^Unit of Immunology and Functional Genomics, Centro Cardiologico Monzino IRCCS, Milan, Italy; ^3^Department of Clinical Sciences and Community Health, University of Milan, Milan, Italy; ^4^Cardiovascular Tissue Bank, Centro Cardiologico Monzino IRCCS, Milan, Italy

**Keywords:** cardiac mesenchymal stromal cells, cardiac ventricles, functional studies, transcriptome, left ventricle, right ventricle

## Abstract

**Background:**

Left ventricle (LV) and right ventricle (RV) are characterized by well-known physiological differences, mainly related to their different embryological origin, hemodynamic environment, function, structure, and cellular composition. Nevertheless, scarce information is available about cellular peculiarities between left and right ventricular chambers in physiological and pathological contexts. Cardiac mesenchymal stromal cells (C-MSC) are key cells affecting many functions of the heart. Differential features that distinguish LV from RV C-MSC are still underappreciated.

**Aim:**

To analyze the physiological differential amount, function, and transcriptome of human C-MSC in LV versus (vs.) RV.

**Methods:**

Human cardiac specimens of LV and RV from healthy donors were used for tissue analysis of C-MSC number, and for C-MSC isolation. Paired LV and RV C-MSC were compared as for surface marker expression, cell proliferation/death ratio, migration, differentiation capabilities, and transcriptome profile.

**Results:**

Histological analysis showed a greater percentage of C-MSC in RV vs. LV tissue. Moreover, a higher C-MSC amount was obtained from RV than from LV after isolation procedures. LV and RV C-MSC are characterized by a similar proportion of surface markers. Functional studies revealed comparable cell growth curves in cells from both ventricles. Conversely, LV C-MSC displayed a higher apoptosis rate and RV C-MSC were characterized by a higher migration speed and collagen deposition. Consistently, transcriptome analysis showed that genes related to apoptosis regulation or extracellular matrix organization and integrins were over-expressed in LV and RV, respectively. Besides, we revealed additional pathways specifically associated with LV or RV C-MSC, including energy metabolism, inflammatory response, cardiac conduction, and pluripotency.

**Conclusion:**

Taken together, these results contribute to the functional characterization of RV and LV C-MSC in physiological conditions. This information suggests a possible differential role of the stromal compartment in chamber-specific pathologic scenarios.

## Introduction

Left and right cardiac chambers retain well-known physiological differences, linked to their diverse embryological origin, hemodynamic environment, function, structure, and cellular composition (Friedberg and Redington, 2014; Penny and Redington, 2016).

Although cardiomyocytes occupy 75% of adult normal myocardial tissue volume, they represent 30–40% of cardiac cells only. The remaining cells are non-myocytes, including smooth muscle cells, endothelial cells, fibroblasts, and mesenchymal stromal cells (Camelliti et al., 2005; Gray et al., 2018; Perbellini et al., 2018). The distribution of these cell populations in the heart is not homogeneous: the myocardium exhibits distinct regional differences that influence heart physiology and disease development (Souders et al., 2009; Pinto et al., 2016). The different embryonic derivation of the cardiac chambers is the main responsible for this heterogeneity (Moorman et al., 2003). Indeed, the LV originates from the first heart field, while the RV, the intraventricular septum, and the outflow tract derive from the second heart field (Black, 2007; Kelly et al., 2014).

No univocal results have been reported about the cellular composition of the adult cardiac chambers (Zhou and Pu, 2016). The main limitations of the previous studies are the challenges in identifying cell type-specific markers and the different quantification techniques. The majority of the existing studies do not consider the LV and RV as separate entities (Banerjee et al., 2007; Pinto et al., 2016; Zhou and Pu, 2016). In addition, due to the difficulties of access to human tissues, several studies were carried out with murine samples (Banerjee et al., 2007; Pinto et al., 2016).

To the best of our knowledge, no studies so far have characterized the quantity and quality of C-MSC in LV and RV separately. C-MSC are a fibroblastoid cell blend, including fibroblasts, progenitor cells, pericytes, and fibrocytes (Souders et al., 2009), characterized by residual multipotency toward mesenchymal lineages (Pittenger et al., 1999). As stated by the International Society for Cellular Therapy (Dominici et al., 2006), C-MSC are defined by the positive expression of CD44, CD105, and CD29 surface antigens, whereas CD14, CD45, CD34, and CD31 hematopoietic and endothelial markers, and HLA-DR, involved in graft-vs.-host disease, are not expressed (Pilato et al., 2018). CD90 is a fibroblast surface marker (Hudon-David et al., 2007) whose expression in C-MSC is variable, due to the heterogeneity in the cell population, only partially represented by fibroblasts. C-MSC can differentiate into several cell types like adipocytes, chondrocytes, and osteoblasts, under standard differentiating conditions *in vitro* (Dominici et al., 2006).

C-MSC exert important functions in the heart in both physiological and pathologic conditions (Brown et al., 2005). They are essential to maintaining myocardial structure integrity and cardiac function, contributing to biochemical, mechanical, and electrical physiology in healthy hearts (Camelliti et al., 2005). The role of C-MSC in many cardiac diseases is increasingly recognized. In injury conditions, they can participate to wound healing and fibrotic remodeling (Long and Brown, 2002; Jugdutt, 2003). In addition, they can undergo adipogenic differentiation in the heart in particular diseases (Abel et al., 2008; Sommariva et al., 2016). Aside from a direct role, C-MSC influence cardiomyocyte function in pathological states (Takeda and Manabe, 2011). Interestingly, an immunomodulatory role of C-MSC has been described (Prockop and Oh, 2012; Czapla et al., 2016; Diedrichs et al., 2019). Moreover, high expectations are raised in the use of C-MSC in regenerative medicine scenarios (Pittenger and Martin, 2004; Bagno et al., 2018; Braunwald, 2018). For these reasons, a better characterization of C-MSC functions and properties may be clinically relevant, both as a target and as a tool for new therapies (Frangogiannis, 2017).

In this work, we describe, for the first time, differences in quantity, distinctive characteristics, functional properties, and resting transcriptome profile of C-MSC obtained from human RV and LV.

## Materials and Methods

Anonymized data and materials have been made publicly available at the NCBI’s GEO repository and can be accessed at https://www.ncbi.nlm.nih.gov/geo/query/acc.cgi?acc=GSE142205.

### Study Patients’ Population

Human hearts are collected during multi-organ explants from heart-beating donors. Those excluded from organ transplantation for technical reasons (microbiological, serological reasons despite normal echocardiographic parameters) are sent to the “Cardiovascular Tissue Bank” of Centro Cardiologico Monzino IRCCS for aortic and pulmonary valve banking. Among the tissues discarded during valve preparation, transmural mid-chamber free wall samples from LV at the anterolateral mid-papillary level and RV at the anterior papillary muscle level, above moderator band insertion, were collected and processed for tissue sections. From six of the enrolled subjects, endocardial–myocardial ventricular tissue from the same origin was collected to isolate C-MSC (Pilato et al., 2018). See Supplementary Figure S1.

Supplementary Table S1 summarizes the clinical features of 13 healthy donors, dead due to accident, enrolled in this study. LV and RV autopsy samples, processed as described above, were obtained from all the enrolled individuals.

### Heart Tissue Section Preparation and Immunofluorescence Analysis

Human ventricular samples were fixed in 4% paraformaldehyde (Santa Cruz) in PBS (Lonza) and processed for paraffin embedding. Paraffin-embedded sections (6 μm thick) were de-waxed in xylene and rehydrated in ascending alcohols. The immunofluorescence analysis was performed following antigen retrieval with incubation with target retrieval solution citrate pH 6/microwave (Dako). Sections were incubated at 4°C overnight with primary antibodies for the detection of mesenchymal surface markers (see Supplementary Table S2), namely, anti-CD29 (1:40; Leica), anti-CD44 (1:200; Abcam), and anti-CD105 (1:100; Abcam) diluted in 2% goat serum (Sigma–Aldrich). After washing with PBS, sections were incubated for 1 h at RT in the dark with proper secondary antibodies (see Supplementary Table S3). Nuclear staining was performed by incubating sections with Hoechst 33342 (1:1000; Life Technologies). Sections were observed by Zeiss Axio Observer.Z1, with Apotome technology, and images acquired with the software AxioVision Rel. 4.8. For each explanted heart patient, five slices and at least 10 fields for each slice were examined.

### C-MSC Isolation and Culture

LV and RV C-MSC were isolated and cultured as previously reported (Sommariva et al., 2016; Pilato et al., 2018). Briefly, LV and RV samples were digested with 3 mg/ml collagenase NB4 (Serva) for 1.5 h under continuous agitation. Each LV and RV tissue sample used for C-MSC obtainment was weighted before the digestion process.

The digested tissue and cells were seeded onto uncoated Petri dishes (Corning) in a growth medium [IMDM supplemented with 20% FBS Hyclone (Euroclone), 10 ng/ml basic fibroblast growth factor (R&D Systems), 10,000 U/ml penicillin (Invitrogen), 10,000 μg/ml streptomycin (Invitrogen), and 20 mmol/l L-glutamine (Sigma–Aldrich)].

After 10 days, isolated C-MSC were detached and counted to determine the number of cells obtained from each sample. The counted number was normalized on the grams of digested tissue.

The medium used to prompt the adipogenic differentiation of C-MSC consists of IMDM supplemented with 10% FBS (Sigma–Aldrich), 0.5 mmol/l 3-isobutyl-1-methylxanthine (Sigma–Aldrich), 1 μmol/l hydrocortisone (Sigma–Aldrich), 0.1 mmol/l indomethacin (Sigma–Aldrich), 10,000 U/ml penicillin (Invitrogen), 10,000 μg/ml streptomycin (Invitrogen), and 20 mmol/L L-glutamine (Sigma–Aldrich).

The medium for the evaluation of collagen production and deposition consists of IMDM supplemented with 2% FBS (Sigma–Aldrich), 10 ng/ml basic fibroblast growth factor (R&D Systems), 10,000 U/ml penicillin (Invitrogen), 10,000 μg/ml streptomycin (Invitrogen), and 20 mmol/l L-glutamine (Sigma–Aldrich).

### Flow Cytometry Analysis

To confirm the mesenchymal lineage of RV and LV C-MSC, cells cultured in the basal medium were detached with TrypLE^TM^ Select Enzyme (Thermo Fisher Scientific), incubated with FITC/APC/PE-conjugated antibodies (see Supplementary Table S2) in 100 μl PBS, and analyzed by flow cytometry (Gallios, Beckman Coulter). The antibodies used are the following: CD29, CD44, CD105, CD90, (mesenchymal markers), CD14, CD31, CD34, CD45 (endothelial and hematopoietic markers), and HLA-DR (immunogenicity marker).

### Cell Growth Analysis

LV and RV C-MSC were plated at a concentration of 10,000 cells/cm^2^ in the growth medium (see section “C-MSC isolation and culture”) in four replicates. After 24, 48, 72, and 96 h, cells were detached and counted to analyze their growth rate.

### Apoptosis and Necrosis Assay

To evaluate apoptosis and necrosis rate in LV and RV C-MSC, Single-Channel Dead Cell Apoptosis Kit with Annexin V Alexa Fluor^TM^ 488 and SYTOX^TM^ Green Dyes (Thermo Fisher Scientific) has been used, according to the manufacturer’s instructions. Briefly, cells were plated at a concentration of 20,000 cells/cm^2^ in the growth medium for 24 h. Then, they were detached using TrypLE^TM^ Select Enzyme (Thermo Fisher Scientific) and incubated with Annexin V Alexa Fluor^®^ 488 and SYTOX^®^ Green for 15 min at RT. The fluorescence emission at 530 nm corresponding to apoptotic and necrotic cells has been measured using flow cytometry. The population was separated into three groups: live cells with a low level of fluorescence, apoptotic cells with moderate fluorescence, and dead cells with high intensity of fluorescence.

To assess the apoptotic rate of LV and RV C-MSC during 5 days of culture, we used the IncuCyte live-cell analysis system (Essen BioScience). Briefly, 10,000 cells/cm^2^ were plated in the growth medium in two replicates. After cell attachment, IncuCyte^®^ Annexin V Green Reagent (Essen BioScience) for apoptosis detection was added to the plates. The IncuCyte analysis system scanned the plates every 2 h for 5 days. The Annexin V count normalized on the percentage of confluence was used to analyze the obtained results.

### Motility Analysis

LV and RV C-MSC motility was assessed by scratch wound assay; 40,000 cells/cm^2^ were plated in the growth medium in three replicates. After cell attachment, wounds were created simultaneously in all wells, using IncuCyte WoundMaker (Essen BioScience). The IncuCyte live-cell analysis system (Essen BioScience) scanned the plate every 2 h for 60 h, and the percentage of the dish area occupied by cells was quantified.

### Adipogenic Differentiation and Oil-Red O Staining

LV and RV C-MSC were plated at a concentration of 20,000 cells/cm^2^ in an adipogenic induction medium for 72 h or for 1 week. Lipid accumulation was tested by ORO staining (Fulka). qRT-PCR and Western blot for PPARγ, FABP4, and PLIN1 (for antibodies, see Supplementary Tables S2, S3—for primers, see Supplementary Table S4) were used to check adipogenic mediator expression. As control, ORO staining was performed also on LV and RV C-MSC plated at a density of 20,000 cells/cm^2^ and cultured in the growth medium.

Cardiac mesenchymal stromal cells were fixed with 4% paraformaldehyde (Santa-Cruz) in PBS and then stained with 1% ORO solution (Fulka) in 60% isopropanol (Sigma–Aldrich) for 1 h. After PBS washes to remove the unbound dye, the images were acquired by Axiovert 200M supplied with Axiocam 503 (Zeiss) in black and white, using phase H, to highlight black lipid depots. The quantification was performed with the software AxioVision Rel. 4.8, evaluating at least 10 fields per sample.

### Quantitative Reverse Transcriptase-Polymerase Chain Reaction (qRT-PCR)

LV and RV C-MSC total RNA extracted using TRIzol Reagent (Thermo Fisher Scientific) was reversely transcribed using SuperScript III Reverse Transcriptase (Invitrogen). Each sample was analyzed in duplicates with each primer pair, using 10 ng of cDNA, with CFX96 Touch Real-Time PCR Detection System (Bio-Rad) using iQ SYBR Green Supermix (Bio-Rad). Threshold cycles were normalized against the expression of the housekeeping gene *GAPDH* (ΔCt). Primer sequences are reported in Supplementary Table S4.

### Western Blot

Total proteins from LV and RV C-MSC were obtained by Cell Lysis Buffer (Cell Signaling). After quantification with DC protein assay (Bio-Rad), proteins were run on SDS-PAGE gel (NuPAGE precast 4–12%, Invitrogen) and transferred to the Trans-Blot^®^ Turbo^TM^ nitrocellulose membrane (Bio-Rad) with the Trans-Blot^®^ Turbo^TM^ transfer system. The membrane was blocked in PBS containing 0.05% Tween^®^ 20 (Sigma–Aldrich) and 5% skimmed milk (ChemCruz) for 1 h at RT and incubated overnight at 4°C with the primary antibodies against GAPDH and the main adipogenic proteins PPARγ, PLIN1, and FABP4 (see Supplementary Table S2). After washes in PBS containing 0.05% Tween^®^ 20 (Sigma–Aldrich), the membranes were incubated 1 h at RT with the appropriate HRP-conjugated secondary antibody (see Supplementary Table S3). Blots were washed and developed with the ECL system (Amersham) and images acquired and quantified with the UVItec Cambridge system. The normalization was performed on the housekeeping protein GAPDH.

### Collagen Production

LV and RV C-MSC were plated at a concentration of 30,000 cells/cm^2^ in the growth medium with a reduced amount of FBS (2%; see section “C-MSC Isolation and Culture”) for 5 days, without medium change. The collagen production and myofibroblast differentiation were assessed through Sircol collagen analysis (Biocolor Life Science Assays), Western Blot, and qRT-PCR. Sircol Collagen Assay was performed on LV and RV C-MSC lysates and supernatants, after their collection in low-protein-binding tubes. The cellular lysates underwent collagen isolation and concentration step overnight. Both C-MSC lysates and supernatants were then mixed with 1 ml of Sircol Dye Reagent at RT for 30 min to ensure the precipitation of collagen. The obtained pellet was dissolved in Alkali Reagent, and the amount of collagen was determined at 540 nm using a microplate reader (Mithras LB 940; Berthold Technologies) and calculated based on a standard curve of soluble collagen.

### RNA-Seq Analysis and Data Processing

Total RNA of 300,000 cultured, amplified C-MSC from LV (*n* = 6) and RV (*n* = 6) was isolated using TRIzol^TM^ Reagent (Thermo Fisher Scientific), precipitated through the ammonium acetate/ethanol method and, then, treated with DNAse (TURBO DNAse; Thermo Fisher Scientific) to remove genomic DNA contamination. The total RNA concentration and quality were assessed, respectively, by micro-volume spectrophotometry on an Infinite M200 PRO Multimode microplate reader (Tecan, Mannedorf, Switzerland) and by microfluidics electrophoresis using the RNA 6000 Nano Assay Kit on the 2100 Bioanalyzer system (Agilent Technologies, Santa Clara, CA, United States). Poly(A)^+^ RNA enrichment was performed using Dynabeads mRNA DIRECT Micro Kit (Thermo Fisher Scientific) starting from 6 μg of total RNA. Barcoded libraries were constructed using Ion Total RNA-Seq Kit v2.0 and Ion Express RNA-Seq Barcode kit (Thermo Fisher Scientific) following the manufacturer’s instructions. Briefly, after poly(A)^+^ RNA fragmentation using RNAse III, hybridization and ligation of barcoded adapters for stranded RNA sequencing were performed, followed by reverse transcription. cDNA fragments of 200 bp of each sample were amplified by 16 cycles of PCR using the specific “Barcode BC primers” for library demultiplexing and quantified on the 2100 Bioanalyzer system (Agilent Technologies, Santa Clara, CA, United States). One hundred pM diluted libraries were randomly pooled (six samples per pool). Templated Ion sphere particles preparation and chip loading were, then, performed by the automated Ion Chef System and Ion 550 Kit-Chef reagents and disposables. Loaded Ion 550 Chips were run on Ion GeneStudio S5 Prime System (all kits and instruments for sequencing were provided by Thermo Fisher Scientific).

Sequential aligning of raw reads was performed against the GRCh38 Human Genome reference (last release) with the most updated version of the “Spliced Transcripts Alignment to a Reference (STAR)” software (Dobin et al., 2013) and with “Bowtie2” (Langmead and Salzberg, 2012) to align locally any reads not mapped by STAR. Gene expression quantification and annotation were computed by “featureCounts” (Liao et al., 2014).

Raw count data were imported into the R software v3.5.0. and filtered to retain genes with a minimum of 10 counts in at least 50% of the samples. Differential expression analysis was performed by a negative binomial GLM approach (using the edgeR/Bioconductor package) (Robinson and Oshlack, 2010; McCarthy et al., 2012) along with the estimation of latent variables, technical batch effects, or biological confounding variables, for adjusting the statistical model (using the RUVSeq R/Bioconductor package) (Risso et al., 2014). The number of K factors was chosen by comparing unadjusted vs. adjusted expression data by the use of diagnostic plots, i.e., relative log expression (RLE) plot, scatter plot of the first two principal components derived from PCA performed on total data, and histogram of the *P*-value distribution for testing the differential expression between LV vs. RV. A *K* = 3 factor of “unwanted variation” showed the best trade-off between data adjustment and the risk of data overcorrection and was, thus, used as covariates for model adjustment in a paired-sample data analysis. Genes were deemed as significantly different for FDR-adjusted *P*-value < 0.05. The reliability of the differential expression analysis results was further assessed by exploring the histograms of the *P*-value distribution, which showed a uniformly flat distribution across the unit interval (null *P*-values) with a peak near zero (*P*-values for alternative hypotheses) (Leek and Storey, 2008).

Functional inference analysis took advantage of prior biological knowledge of genes grouped by pathways and used for GSEA (software v4.0) (Subramanian et al., 2005). Gene sets of various pathway repositories were retrieved as a unique, merged Gene Matrix Transposed file format (^∗^.gmt) from the Bader Lab gene-set collections^1^ to perform a single GSEA run. A combined gene rank score (cs) was applied to weigh the relevance of the genes by taking into consideration both the magnitude [i.e., log2 fold change (FC)] and the statistical score of the gene expression differences [likelihood ratio (LR)] and was used as the gene-ranking metric for the GSEA pre-ranked tool option. Other GSEA parameters included 10,000 permutations and gene-set size limit ranging from 10 to 250 genes. To reduce redundancy and highlight grouping of functionally related gene sets, GSEA results were visualized through an enrichment network of the most significant pathways (FDR q-value < 0.05) with the Enrichment Map Software v.3.2.1 (Merico et al., 2010), implemented as a plug-in in the Cytoscape v.3.7.1 platform (Shannon et al., 2003).

### Statistical Analysis

Continuous variables are reported as mean ± standard error. Comparisons between groups were performed using two-tailed paired Student’s *t*-test. Dissimilarities in the growth rate between LV and RV C-MSC were evaluated by testing the difference between the two linear regression slopes with the following method: *t* = (b1-b2)/sb1,b2 where b1 and b2 are the two slope coefficients and sb1,b2 the pooled standard error of the slope. To test if the distribution of the migration curves of LV and RV cells was diverse, we performed a Kolmogorov–Smirvov test, followed by fitting analyses with linear and quadratic regression models. Statistics were performed using GraphPad Prism 5 software. Results were considered statistically significant for *P*-values < 0.05.

## Results

### Quantitative Analysis of C-MSC in LV and RV Tissues

**FIGURE 1:**
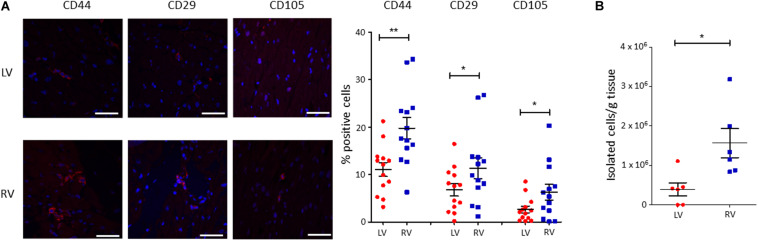
A higher amount of cardiac mesenchymal stromal cells (C-MSC) is present in the RV when compared to the left one. **(A)** Immunofluorescence staining on human left ventricular (LV) and right ventricular (RV) total tissue for the mesenchymal markers CD29, CD44, and CD105. On the left, representative images are shown. The red staining is relative to the mesenchymal markers, the blue signal marks the nuclei (Hoechst 33342). On the right, the quantification of the percentage of positive cells is reported. The scale bar indicates 50 μm. *n* = 13 each; *p < 0.05 (paired *t*-test). **(B)** C-MSC were isolated from either LV or RV human samples. A higher amount of C-MSC was obtained from RV samples. *n* = 6 each; *p < 0.05, **p < 0.01 (paired *t*-test).

To characterize the amount of C-MSC in the two cardiac ventricles, we analyzed LV and RV serial slices from 13 healthy donors (see Supplementary Table S1 for donor characteristics) for CD44, CD29, and CD105 mesenchymal marker expression. RV presented a higher percentage of positive cells compared with LV (*n* = 13; % LV CD44^+^ cells 11.05 ± 1.450 vs. % RV CD44^+^ cells 19.75 ± 2.210, *P* = 0.001; % LV CD29^+^ cells 6.764 ± 1.285 vs. % RV CD29^+^ cells 11.31 ± 2.178, *P* = 0.020; % LV CD105^+^ cells 2.638 ± 0.7078 vs. % RV CD105^+^ cells 6.269 ± 1.627, *P* = 0.011; Figure 1A).

We then proceeded with a quantitative evaluation of C-MSC isolated from LV and RV tissues through the digestion procedure, already described in Pilato et al. (2018). A significantly greater amount of RV C-MSC has been obtained from the same quantity of source tissue (*n* = 6; LV C-MSC 396,047 ± 165,909 vs. RV C-MSC 1564,440 ± 366,220; *P* = 0.040; Figure 1B), in line with the physiological higher number of C-MSC in the RV (Figure 1A).

### Immuno-Phenotyping of Isolated LV and RV C-MSC

**FIGURE 2:**
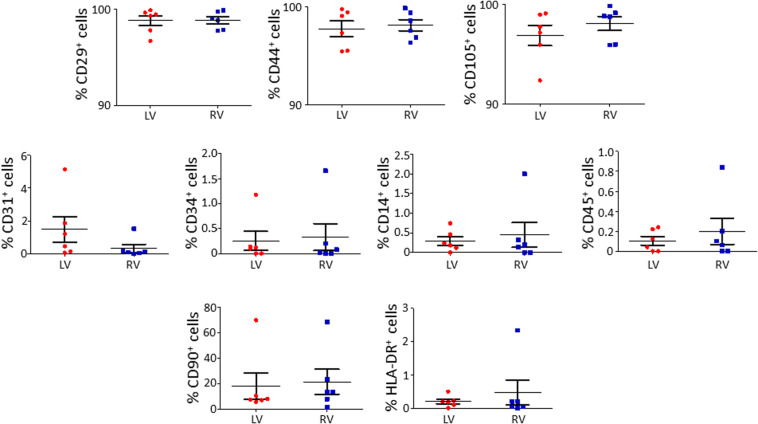
The isolated left ventricle (LV) and right ventricle (RV) cardiac mesenchymal stromal cells (C-MSC) are not different for surface marker expression. LV and RV C-MSC were characterized for surface marker expression, showing no differences. Mesenchymal markers CD29, CD44, and CD105, endothelial markers CD31 and CD34, hematopoietic markers CD14 and CD45, the fibroblast marker CD90, and the alloreactivity marker HLA-DR have been used. *n* = 6 each (paired *t*-test).

We characterized the obtained cells for surface marker expression (Figure 2; please see Supplementary Table S2 for the list of used antibodies). Both LV and RV C-MSC were near 100% positive for the mesenchymal markers CD44, CD29, and CD105, whereas they displayed negligible values for CD14, CD45, CD34, and CD31 markers, which were assessed to exclude hematopoietic and endothelial cell contamination. Moreover, HLA-DR, a marker of alloreactivity, was not detected in either LV or RV cells. The percentage of CD90^+^ cells was measured to define the number of fibroblasts in the heterogeneous population of C-MSC (Hudon-David et al., 2007). As shown in Figure 2, the percentage of CD90^+^ cells was comparable in the two populations. In conclusion, for all the surface markers screened, we found a similar pattern of expression in LV and RV C-MSC, indicating no differences in the mesenchymal identity of the two populations (see Supplementary Table S5).

### Functional Analysis on LV and RV C-MSC

#### Growth Rate of LV and RV C-MSC

**FIGURE 3:**
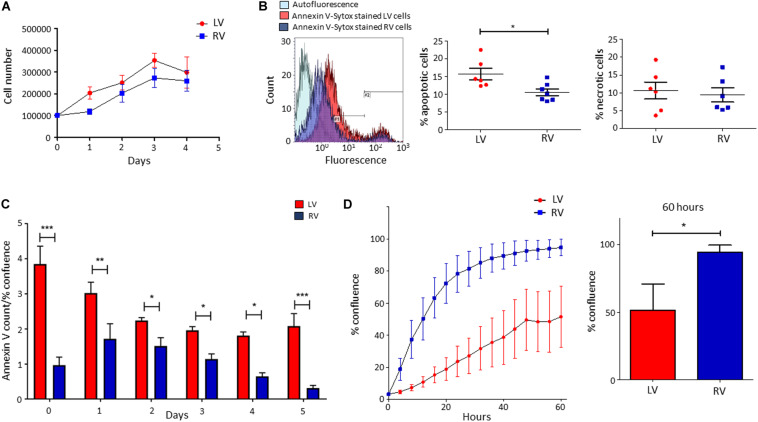
Higher apoptosis in growth conditions characterizes left ventricle (LV) cardiac mesenchymal stromal cells (C-MSC) with respect to right ventricle (RV) cells. RV C-MSC show greater motility when compared to LV cells. **(A)** The growth curves of LV and RV C-MSC in the culture medium are reported. Cells were cultured for 4 days, and the number of cells was counted every 24 h. No differences in growth curve slopes have been obtained. *n* = 6 each (linear regression slope comparison). **(B)** The percentage of apoptotic and necrotic cells was evaluated in the culture medium. In the left panel, representative flow cytometry histograms comparing LV and RV C-MSC patterns are shown. As shown in the right graphs, a higher percentage of apoptotic (measured in #1 interval) LV C-MSC than RV cells has been found. No differences between the necrotic (measured in #2 interval) cell amount were recorded. *n* = 6 each; *p < 0.05 (paired *t*-test). **(C)** The apoptosis rate of LV and RV C-MSC for different time-points is reported. Cells were cultured for 5 days in growth medium. The Annexin V count normalized on the percentage of confluence is shown. For all the time-points, a trend of higher apoptosis was measured in LV cells, significantly different from RV C-MSC apoptotic rate at days 0, 1, 2, 3, 4, and 5. *n* = 6 each; *p < 0.05, **p < 0.01, ***p < 0.001 (two-way ANOVA). **(D)** The mean values of confluence percentage of LV and RV cells during scratch wound assay are reported (left panel). Higher motility of RV C-MSC, if compared to LV cells, has been found. Cell confluence was recorded for 60 h. The right graph depicts the difference in confluence percentage at 60 h. *n* = 6 each; *p < 0.05 (paired *t*-test).

We performed growth curves of LV and RV C-MSC in culture medium for 4 days, up to growth plateau achievement. As shown in Figure 3A, the comparison between the growth curve slopes revealed that both LV and RV C-MSC have a similar growth trend (*n* = 6; *P* = 0.67).

#### Cell Death of LV and RV C-MSC

We evaluated the number of apoptotic and necrotic cultured LV and RV C-MSC. Figure 3B shows that LV cells presented a higher percentage of apoptotic cells if compared with RV C-MSC (*n* = 6; LV C-MSC 15.66 ± 1.62 vs. RV C-MSC 10.91 ± 1.035%; *P* = 0.049), while no differences in marker of necrosis were found between the two cell populations (*n* = 6; LV C-MSC 10.56 ± 2.37 vs. RV C-MSC 9.43 ± 1.98%; *P* = 0.711).

Basing on this result, we followed LV and RV C-MSC with a live-imaging technique for 5 days in culture conditions and we assessed their apoptotic rate. Figure 3C shows that LV C-MSC presented a higher apoptosis rate if compared with RV cells at all time-points, confirming Figure 3B results.

#### LV and RV C-MSC Motility

By performing the scratch wound assay, we assessed the migration capability of LV and RV C-MSC. As reported in Figure 3D, the percentage of confluence detected in the two cell populations during the 60 h of measurements revealed a different migration rate in LV and RV cells, with a significant discrepancy in the distributions (*P* < 0.001). In particular, for LV cells, the distribution was linear (*R*^2^ = 0.98), whereas, for RV cells, the distribution was not linear (*R*^2^ = 0.79) but fitted a quadratic curve (*R*^2^ = 0.98). In addition, RV C-MSC reached a significantly higher percentage of confluence if compared with LV cells at 60 h (*n* = 6; LV C-MSC 51.56 ± 19.15 vs. RV C-MSC 94.64 ± 5.17%; *P* = 0.046).

#### LV and RV C-MSC Adipogenic Differentiation

**FIGURE 4:**
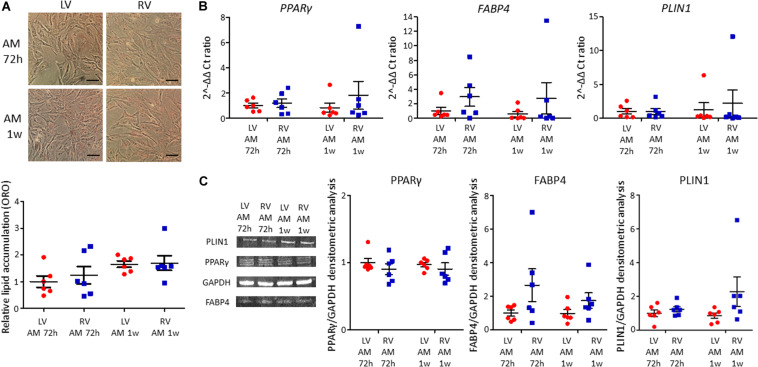
No differences in adipogenic differentiation between left ventricle (LV) and right ventricle (RV) cardiac mesenchymal stromal cells (C-MSC) have been observed. **(A)** The upper panels show Oil Red O staining representative images of LV and RV C-MSC cultured for 72 h and 1 week in the adipogenic medium (AM). The scale bar indicates 50 μm. The quantification of cell lipid accumulation is provided in the bottom panel. *n* = 6 each (paired *t*-test). **(B)** qRT-PCR analysis of *PPAR*γ, *FABP4*, and *PLIN1* expression in LV and RV C-MSC cultured for 72 h and 1 week in AM, normalized on the housekeeping gene *GAPDH*. 2^–ΔΔ^ Ct ratio is shown: 2^–ΔΔ^ Cts of each group are normalized on 2^–ΔΔ^ Ct values of LV C-MSC cultured in AM for 72 h. *n* = 6 each (paired *t*-test). **(C)** The protein extracts of LV and RV C-MSC cultured for 72 h and 1 week have been analyzed by Western blot. The densitometric analysis of PPARγ, FABP4, and PLIN1 normalized on the housekeeping protein GAPDH is shown. *n* = 6 each (paired *t*-test).

LV and RV C-MSC were cultured in adipogenic conditions for 72 h or 1 week, to understand their capability to accumulate lipids and differentiate in adipocytes. The ORO staining, which quantifies the intracellular neutral lipid accumulation, revealed similar lipid accumulation between LV and RV C-MSC, both after 72 h (*n* = 6; relative lipid accumulation LV C-MSC 1.00 ± 0.21 vs. RV C-MSC 1.24 ± 0.33; *P* = 0.754; Figure 4A) and 1 week (*n* = 6; relative lipid accumulation LV C-MSC 1.66 ± 0.12 vs. 1.70 ± 0.28; *P* = 0.99; Figure 4A). In agreement with the comparable levels of lipid accumulation between LV and RV cells, also the expression of the adipogenic genes *PPAR*γ, *FABP4*, and *PLIN1* were similar in the two cell populations at the considered time-points (Figure 4B and Supplementary Table S6). Moreover, the correspondent adipogenic proteins showed analogous levels (Figure 4C and Supplementary Table S6). As control, we performed the ORO staining also in LV and RV C-MSC cultured in the growth medium, obtaining a very small amount of lipid accumulation and no differences in the two cell populations (Supplementary Figure S2).

#### LV and RV C-MSC Collagen Production and Deposition

**FIGURE 5:**
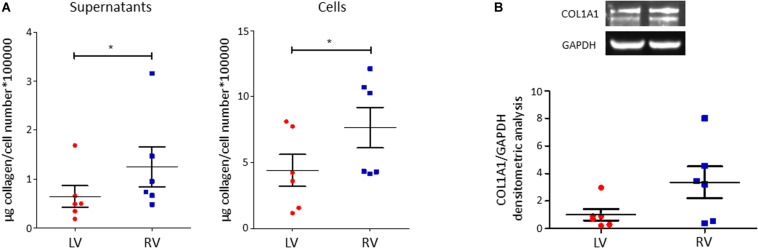
Right ventricle (RV) cardiac mesenchymal stromal cells (C-MSC) produce more collagen than left ventricle (LV) cells. **(A)** Collagen production quantification with Sircol assay in supernatants (left graph) and cells (right graph) of LV and RV C-MSC cultured for 5 days in the basal medium with 2% serum. As shown in the graphs, RV cells produce more collagen than LV cells. *p < 0.05; *n* = 6 each (paired *t*-test). **(B)** Western blot analysis of COL1A1, the more expressed collagen type, confirmed Sircol result. A trend of higher expression of COL1A1 was evaluated in RV C-MSC. *n* = 6 each (paired *t*-test).

C-MSC are known to produce collagen. Comparing LV and RV cells, we found a higher collagen production in RV cells after 5 days of culture, both evaluating total collagen quantity in supernatants (left panel LV C-MSC 0.64 ± 0.22 μg collagen/cell number^∗^100,000 vs. RV C-MSC 1.24 ± 0.41 μg collagen/cell number^∗^100,000; *P* = 0.04; Figure 5A) and in the deposited extracellular matrix (right panel LV C-MSC 4.41 ± 1.21 vs. RV C-MSC 7.65 ± 1.54 μg collagen/cell number^∗^100,000; *P* = 0.05; Figure 5A). On protein lysates, we evaluated the levels of the more expressed collagen type, COL1A1, normalized on the housekeeping protein GAPDH, finding a trend of increased expression in RV cells, according with the analysis of total collagens (LV C-MSC 1.00 ± 0.41 vs. RV C-MSC 3.36 ± 1.15; *P* = 0.11; Figure 5B).

### Transcriptomic Analysis of LV and RV C-MSC

To extend the characterization of LV and RV C-MSC, we performed transcriptomic profiling in resting conditions.

Following data processing and raw count filtering, we identified 14,486 expressed genes, which include 11,942 protein-coding genes, 1754 pseudogenes, 768 long non-coding genes, and 22 short non-coding genes (Supplementary Figure S3; see annotation in Supplementary File S1 for details).

**FIGURE 6:**
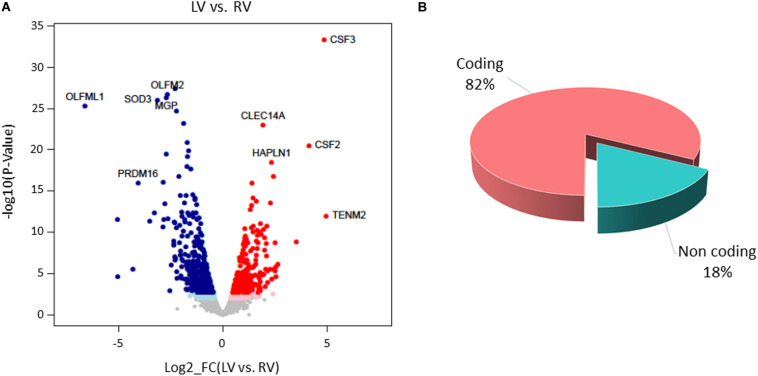
Differential gene expression between left ventricle (LV) and right ventricle (RV) cardiac mesenchymal stromal cells (C-MSC). **(A)** Scatterplot of the log2 fold change (FC) vs. the significance (*x*- and *y*-axes, respectively) for the paired comparison of LV vs. RV C-MSC. Red and blue dots represent genes overexpressed in LV and RV C-MSC, respectively. Light red and light blue dots are genes significant at the nominal *P*-value < 0.01, whereas red and blue dots represent significant differentially expressed (DE) genes that withstood adjustment for multiple testing (adjusted *P*-value < 0.05). Ten of the top DE genes with the highest combined rank score (the product of the log2 FC × likelihood ratio) are shown. **(B)** Pie chart of the percentage of coding (pink) and non-coding (light blue) differentially expressed genes; more than 80% of DE genes are protein coding.

Paired-sample analysis and adjustment for confounding “latent” variables allowed reducing the effects of heterogeneity among subjects, thus unveiling specific changes between LV vs. RV. We detected 652 DE genes with log2 FCs ranging from −6.6 to 4.9 at FDR < 0.05. Among them, 271 genes presented higher expression levels in LV and 381 in RV samples (Figure 6 and Supplementary File S1). The histogram of *P*-value distribution confirmed the reliability of differential expression (DE) analysis (Supplementary Figure S4).

**FIGURE 7:**
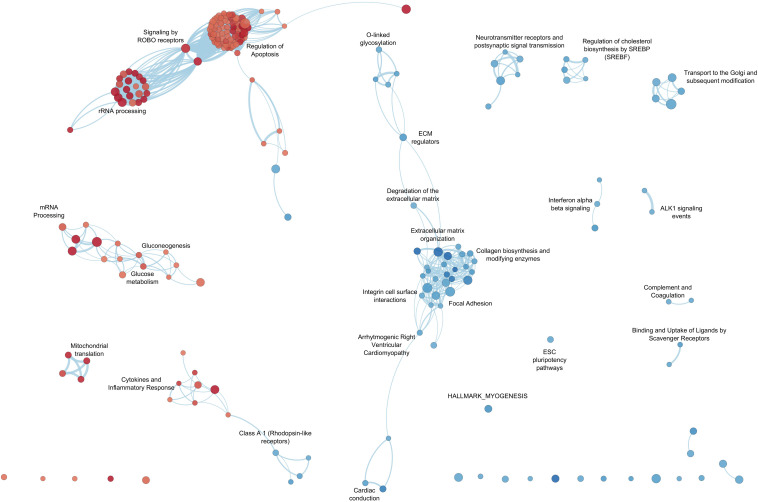
Enrichment map for left ventricle (LV) and right ventricle (RV) cardiac mesenchymal stromal cells (C-MSC). The enrichment network shows the pathway gene sets (nodes) that are significantly associated either with LV or RV ventricles (false discovery rate < 0.05). Node color refers to the association with the phenotype (LV = red, RV = blue); node gradient color is proportional to the gene-set normalized enrichment score (NES), from lower (light) to higher (dark); node size is proportional to the gene-set size. Edges connect related pathways. Edge thickness is proportional to the similarity between two pathways, for a cutoff = 0.25 of the combined Jaccard plus overlap coefficient.

By GSEA, we identified a considerable number of significant pathways that characterize LV or RV C-MSC. To facilitate result interpretation and visualize the relationships among the most significant gene sets, we drew an enrichment network of GSEA results for the paired comparison between LV vs. RV (Figure 7). The most representative pathways associated with LV are suggestive for mRNA and rRNA processing, signaling by ROBO receptors, regulation of apoptosis, glucose metabolism, mitochondrial translation, and cytokine and inflammatory response. Conversely, the most representative pathways associated with RV (and negatively associated with LV) were related to extracellular matrix organization; collagen biosynthesis; integrin cell surface interactions; cardiac conduction; regulation of cholesterol biosynthesis by SREBP (SREBF); binding and uptake of ligands by scavenger receptors; arrhythmogenic right ventricular cardiomyopathy; GPCR Class A 1 rhodopsin-like receptor, neurotransmitter receptor and postsynaptic signal transmission; and interferon-alpha and beta signaling. Overall, these findings suggest profound differences in the transcriptional programs involved in remodeling, energy metabolism, responses to cytokines or inflammatory stimuli, electrical conduction, pluripotency, repair, and regeneration between LV and RV C-MSC.

## Discussion

To date, the literature on human cardiac cell composition provides few and conflicting data (Zhou and Pu, 2016). Despite the increasingly recognized importance of the non-myocyte compartment (Tian and Morrisey, 2012), the C-MSC population has not been previously in-depth investigated in this regard. In addition, C-MSC differential role within the cardiac chambers, with particular regard to the left and right human ventricles, is underinvestigated. A more robust definition is required to distinguish subsets of stromal cells with specialized functions in diverse tissues. In fact, due to morphology, immunophenotype, and differentiation potential similarity, the nomenclature of MSC and fibroblasts is often used indistinctively (Hematti, 2012) to name the same cell type isolated with the same method (Rockel et al., 2019).

In this study, by using samples obtained from cadaveric donors, we performed a better characterization of human C-MSC quantity, distinctive transcriptional configuration, and functional properties, focusing on differences related to the two cardiac ventricular chambers.

Although all of the cardiac samples used for this study were obtained following a reproducible procedure, using proper references to ensure the collection of comparable samples, a residual heterogeneity between samples cannot be excluded, comprising imbalanced representation of gender.

The immunofluorescence analysis of human cardiac left and right ventricular tissues showed a higher percentage of cells positive for the mesenchymal markers CD29, CD44, and CD105 in the RV. In general, the total number of C-MSC resulted proportionally low in both ventricles. The majority of non-myocytes was previously thought to be fibroblasts (Camelliti et al., 2005). However, this information has been recently questioned by Pinto et al. (2016). Indeed, the authors demonstrate that endothelial cells outnumber the other non-myocyte cardiac cell types in the adult ventricles, representing >60% of cells. Specifically, fibroblasts accounted for less than 20% of the non-myocytes. Overall, our results concur with this evidence and add a clear definition of the differential C-MSC abundance in the two ventricular chambers.

In accordance with tissue analysis, a higher amount of C-MSC can be isolated from the human RV than from the left one. Although RV or LV C-MSC have been used and characterized alternatively (Chong et al., 2013; Czapla et al., 2016; Sommariva et al., 2016; Le et al., 2018, 2019), no previous study performed a direct comparison of cells obtained from the two ventricles of the same individual. This novel approach is useful for a better C-MSC characterization in the human heart. Although the sample size used for *in vitro* experiments is relatively low, it is sufficient to ensure a good statistical power of the analyses, as in a paired-sample design the effects of heterogeneity among subjects are reduced.

Noteworthily, isolated LV and RV C-MSC showed comparable expression of surface markers, despite important differences unveiled with other assays. This indicates, as previously observed (Lv et al., 2014), that surface markers are not sufficient to determine the functional property potential and transcriptomic configuration of C-MSC.

Remarkable concordance was found between functional assays and transcriptome results. No significant difference was detected in C-MSC growth rate. Indeed, no pathway associated with cell growth or proliferation was specifically enriched in LV or RV C-MSC. Instead, a higher apoptosis rate was found in LV C-MSC, in accordance with the fact that LV C-MSC transcripts resulted to be enriched in genes associated with pathways of apoptosis regulation. Moreover, RV cells showed higher motility by scratch wound assay. This is in line with RV C-MSC enrichment of pathways related to integrins, which allow adhesion to promote cell traction (Huttenlocher and Horwitz, 2011). No differences were found between LV and RV C-MSC upon stimulation for adipogenic differentiation, while fibrosis and collagen production were higher in RV C-MSC compared to LV cells. Both results are in line with the transcriptome analysis, in which extracellular matrix organization and collagen production genes were found significantly upregulated in the RV. Interestingly, in healthy hearts, exercise triggers RV profibrotic remodeling (La Gerche et al., 2012).

Furthermore, LV C-MSC transcriptome revealed enrichment in genes associated with cytokines and inflammatory response pathways. C-MSC from the RV are instead enriched in nodes linked to innate immunity mechanisms, such as complement and interferon type 1 signaling pathways. In fact, C-MSC can both amplify inflammatory stimuli and act as anti-inflammatory mediators (Smith et al., 1997; McGettrick et al., 2012; Coulson-Thomas et al., 2016). In this regard, the differential potential of C-MSC from the two chambers in eliciting either innate or cytokine-mediated inflammatory response has never been described and could be of importance for the substrate response to regenerative therapy (Vagnozzi et al., 2019).

A distinctive feature of the LV is the high workload environment. It should not, therefore, be surprising to observe an LV-specific enrichment for pathways linked to energy production and use, such as those related to mitochondria and glucose metabolism (Stanley et al., 2005; Pham et al., 2019).

As expected, given the embryologic origin and the developmental program of the RV (Clapham et al., 2019), we found in RV C-MSC a significant upregulation of the transcription factor *MEF2C*. Moreover, the antisense long-non-coding HAND2-AS1 is specifically more expressed in the LV, pointing to an RV-associated gene downregulation in LV determination (Tsuchihashi et al., 2011).

Taken together, these results highlight relevant physiological differences between LV and RV C-MSC.

In light of this information, the design of new targeted therapeutic strategies to promote heart repair and regeneration could be reconsidered (Pinto et al., 2016). MSC represent a promising tool in the field of regenerative medicine for their therapeutic potential (Pittenger and Martin, 2004; Karantalis and Hare, 2015; Bagno et al., 2018; Braunwald, 2018). Their beneficial properties have been attributed to their capability to migrate to injured areas eliciting immunomodulatory function, to their multipotency, and to their secretion of bioactive compounds (e.g., cytokines, chemokines, growth factors) inducing repair of damaged tissues (Pittenger and Martin, 2004; Caplan and Dennis, 2006; Karantalis and Hare, 2015; Czapla et al., 2016; Bagno et al., 2018). Only few clinical studies, to date, have focused on cardiac-derived MSC, due to the critical access to human cardiac specimens (Miteva et al., 2011; Chugh et al., 2012; Malliaras et al., 2014; Czapla et al., 2016; Detert et al., 2017; Sanz-Ruiz et al., 2017). However, the beneficial effects of cells obtained from the heart are deemed stronger than those obtained using mesenchymal cells from other sources (Rossini et al., 2010; Czapla et al., 2016). Our data on RV enrichment in genes associated with pluripotency allow us to speculate about a greater potential of RV C-MSC in cardiac regeneration.

Moreover, C-MSC are involved in several cardiac conditions. Understanding the healthy state of the human heart, with particular regard to the dissection of cell component properties, may offer a new perspective to heart diseases, which remain the leading cause of death worldwide (Doll et al., 2017). Several diseases differentially affect the two heart chambers. Since determinants of the preferential involvement of LV vs. RV are still unknown, this work may add clues to understand the relative contribution of the stromal compartment.

In particular, our results are of interest for arrhythmogenic cardiomyopathy (ACM), where the role of C-MSC in disease pathogenesis has been increasingly recognized by our group and others (Lombardi et al., 2016; Sommariva et al., 2016). Indeed, the transcriptome analysis showed in RV C-MSC an association with the ACM pathway. For ACM vs. control C-MSC differential transcriptomics, see Rainer et al. (2018).

Pulmonary hypertension is another example of a disease with fibrotic drift mostly affecting the RV (Egemnazarov et al., 2018; Andersen et al., 2019). On top of the anatomical proximity of the trigger, leading to RV pressure overload, the RV maladaptive fibrotic remodeling may partly depend on C-MSC number and specific characteristics. In addition, various arrhythmic diseases, such as Brugada syndrome, ACM, right ventricular outflow tract tachycardia, and Uhl’s anomaly are hallmarked by arrhythmias originating preferentially from the RV (Hoch and Rosenfeld, 1992). Accordingly, we found that RV C-MSC associate with “cardiac conduction” pathways, which include many ion channel genes (Di Resta and Becchetti, 2010). We speculate about the potential involvement of C-MSC in contributing to the altered RV electrical environment. Indeed, a regional difference (RV vs. LV) in current handling is known for cardiomyocytes (Kondo et al., 2006). Given cardiomyocyte–stromal cell coupling (Nattel, 2018), a contribution of stromal cells in RV arrhythmia predisposition cannot be excluded.

Similarly, a contribution of C-MSC characteristics cannot be excluded in disease with preferential LV involvement. An example is constituted by cardiomyopathies of genetic origin, which develop mainly in the LV, while RV dysfunction is an expression of advanced disease progression (Merlo et al., 2016). These inherited cardiomyopathies also involve metabolic and mitochondrial abnormalities (Sacchetto et al., 2019), in agreement with our data showing LV C-MSC enrichment in mitochondrial-associated pathways. Accordingly, multi-organ diseases caused by mitochondrial mutations prevalently cause LV non-compaction cardiomyopathy or LV dilated cardiomyopathy (Towbin and Jefferies, 2017).

## Conclusion

We found that RV and LV differ for quantity and quality of C-MSC. We speculate that these findings may have pathophysiological implications in different areas. Appropriate LV vs. RV C-MSC can be used in disease modeling. Similarly, tissue engineering could benefit from the origin-correspondent C-MSC and gain from our description of cell composition percentage. Regenerative medicine and pharmacological screening, using C-MSC, may take advantage of a responsible choice of the appropriate cell product or derivative, either from the LV or the RV, depending on the application. Moreover, the awareness of the baseline LV or RV C-MSC differences can contribute to a proper understanding of chamber-specific diseases, where C-MSC possibly contribute to regional phenotypic disease expression.

## Data Availability Statement

The dataset has been deposited to the GEO-GSE142205.

## Ethics Statement

This study was approved and reviewed by the Ethics Committee of the IRCCS Istituto Europeo di Oncologia e Centro Cardiologico Monzino (R1020/19-CCM1072). Autoptic donor cardiac samples were obtained from the “Cardiovascular Tissue Bank” of Centro Cardiologico Monzino IRCCS (MTA signed 5/11/2019). Donor heart tissue was collected only after signature of informed consent by relatives, authorizing transplantation and research on the remaining tissue, not suitable for human clinical use.

## Author Contributions

IS, ES, and GiuP conceived the study. IS isolated the cells and performed the experiments to characterize differential C-MSC functions. AS performed immunofluorescence on tissue samples. CV, LP, MC, and GC performed and supervised sequencing and transcriptome analysis. BM, AG, and GiaP provided samples from cadaveric donors and collected clinical data. All authors critically reviewed the manuscript.

## Conflict of Interest

The authors declare that the research was conducted in the absence of any commercial or financial relationships that could be construed as a potential conflict of interest.

## Footnotes

1 http://download.baderlab.org/EM_Genesets/

## Supplementary Material

FIGURE S1 Methodological approach. Transmural samples of left (LV) and right (RV) ventricles have been obtained from mid-chamber free walls of LV at the anterolateral mid-papillary level and of RV at the anterior papillary muscle level, above moderator band insertion. From these samples, total tissue was embedded for immunofluorescence analysis and endocardial–myocardial tissue from the same origin was collected to obtain C-MSC.

FIGURE S2 Lipid accumulation in growth conditions. The left panels show Oil Red O staining representative images of left (LV) and right (RV) cardiac mesenchymal stromal cells (C-MSC) cultured in growth medium (GM). The scale bar indicates 50 μm. The quantification of cell lipid accumulation in comparison to the results obtained in adipogenic medium is provided in the right panel. *n* = 6 each (paired *t*-test).

FIGURE S3 Distribution of gene expression. **(A)** Density distribution of the gene expression levels, grouped by coding (pink) and non-coding (light blue) expressed genes. The protein-coding genes show a higher average expression value than non-coding genes. **(B)** Pie chart of the percentage of coding (pink) and non-coding (light blue) expressed genes; more than 80% of expressed genes are protein-coding.

FIGURE S4 Histogram *P*-value. The histogram of *P*-values distribution for non-DE genes is ideally uniformly distributed across the unit interval, whereas the *P*-values for DE genes present a spike near zero.

TABLE S1 Summary of the clinical features of the healthy controls from which LV and RV samples were obtained.

TABLE S2 Primary antibodies.

TABLE S3 Secondary antibodies.

TABLE S4 Primers.

TABLE S5 Detailed FACS analysis of LV and RV C-MSC.

TABLE S6 Summary of gene and protein expression during LV and RV C-MSC adipogenic differentiation for 72 h and 1 week. 2^–ΔΔ^Ct ratio is reported for qRT-PCR analysis. The densitometric analysis for each protein normalized on GAPDH expression is shown for WB data.

FILE S1 **(a)** Differential gene expression analysis in pair-wise left vs. right ventricle samples. The list shows annotations and statistics for all the genes detected by transcriptome RNA-Sequencing (IonS5, Thermo Fisher Scientific). Significant comparisons for an adjusted *P*-value < 0.05 are highlighted in green. Combined rank-score is calculated as the log2 fold-change (logFC) × LR. **(b)** Gene-set enrichment analysis (GSEA) using AllPathways on combined rank score of pair-wise differential expression analysis. The table lists AllPathways Gene Set Name, Database ID terms, and GSEA statistics for LEFT vs. RIGHT ventricles. Significant tests for a false discovery rate (FDR) < 0.05 are highlighted in green. **(c)** Legend_DE_analysis and Legend_GSEA.

## Abbreviations

C-MSC: cardiac mesenchymal stromal cells
DE: differentially expressed
FABP4: fatty acid-binding protein 4
FBS: fetal bovine serum
FDR: false discovery rate
GAPDH: glyceraldehyde 3-phosphate dehydrogenase
GLM: generalized linear model
GSEA: Gene Set Enrichment Analysis
IMDM: Iscove’s Modified Dulbecco’s Medium
LV: left ventricle
ORO: Oil Red O
PBS: phosphate-buffered saline
PCA: principal component analysis
PLIN1: perilipin-1
PPAR γ: peroxisome proliferator-activated receptor gamma
qRT-PCR: quantitative reverse transcription polymerase chain reaction
RT: room temperature
RV: right ventricle
vs.: versus.
