# Supplementary figures and images for: Human Cardiac Mesenchymal Stromal Cells From Right and Left Ventricles Display Differences in Number, Function, and Transcriptomic Profile
Source: Front Physiol. 2020 Jun 24;11:604. doi: 10.3389/fphys.2020.00604 (PMC7327120; doi:10.3389/fphys.2020.00604)

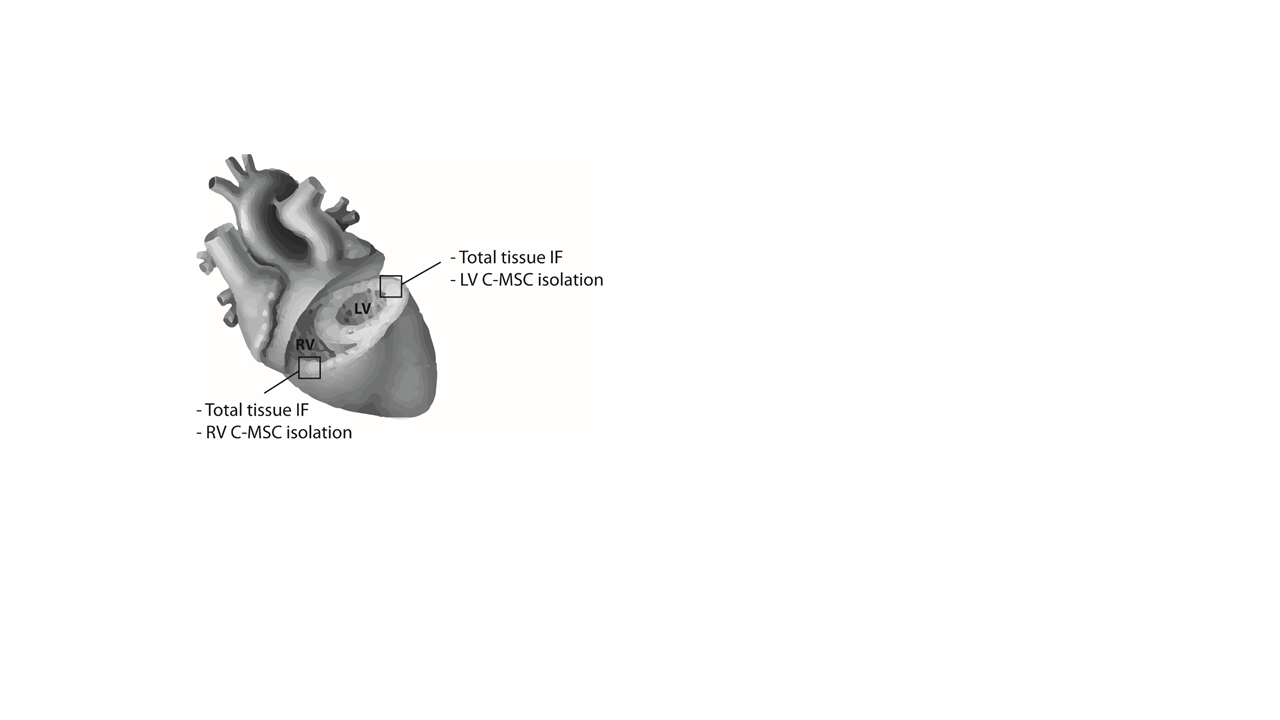

Supplement: FIGURE S1 — Methodological approach. Transmural samples of left (LV) and right (RV) ventricles have been obtained from mid-chamber free walls of LV at the anterolateral mid-papillary level and of RV at the anterior papillary muscle level, above moderator band insertion. From these samples, total tissue was embedded for immunofluorescence analysis and endocardial–myocardial tissue from the same origin was collected to obtain C-MSC. [file Image_1.TIF]

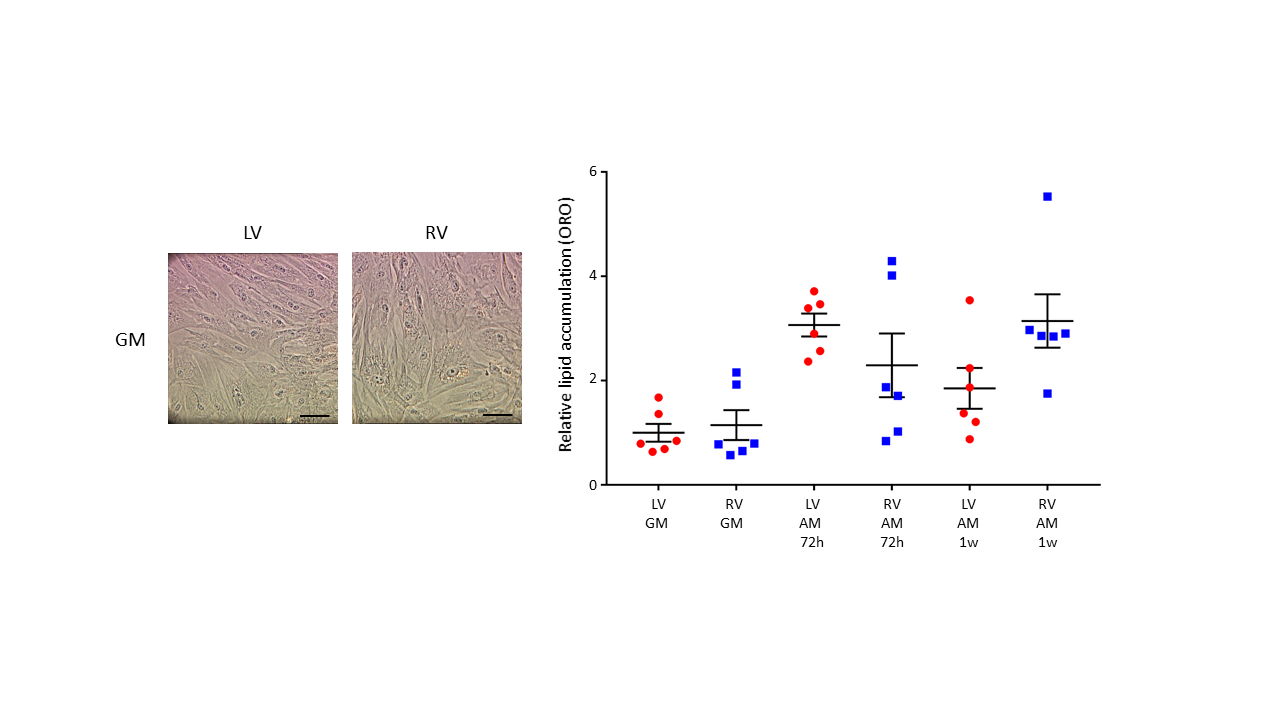

Supplement: FIGURE S2 — Lipid accumulation in growth conditions. The left panels show Oil Red O staining representative images of left (LV) and right (RV) cardiac mesenchymal stromal cells (C-MSC) cultured in growth medium (GM). The scale bar indicates 50 μm. The quantification of cell lipid accumulation in comparison to the results obtained in adipogenic medium is provided in the right panel. n = 6 each (paired t-test). [file Image_2.TIF]

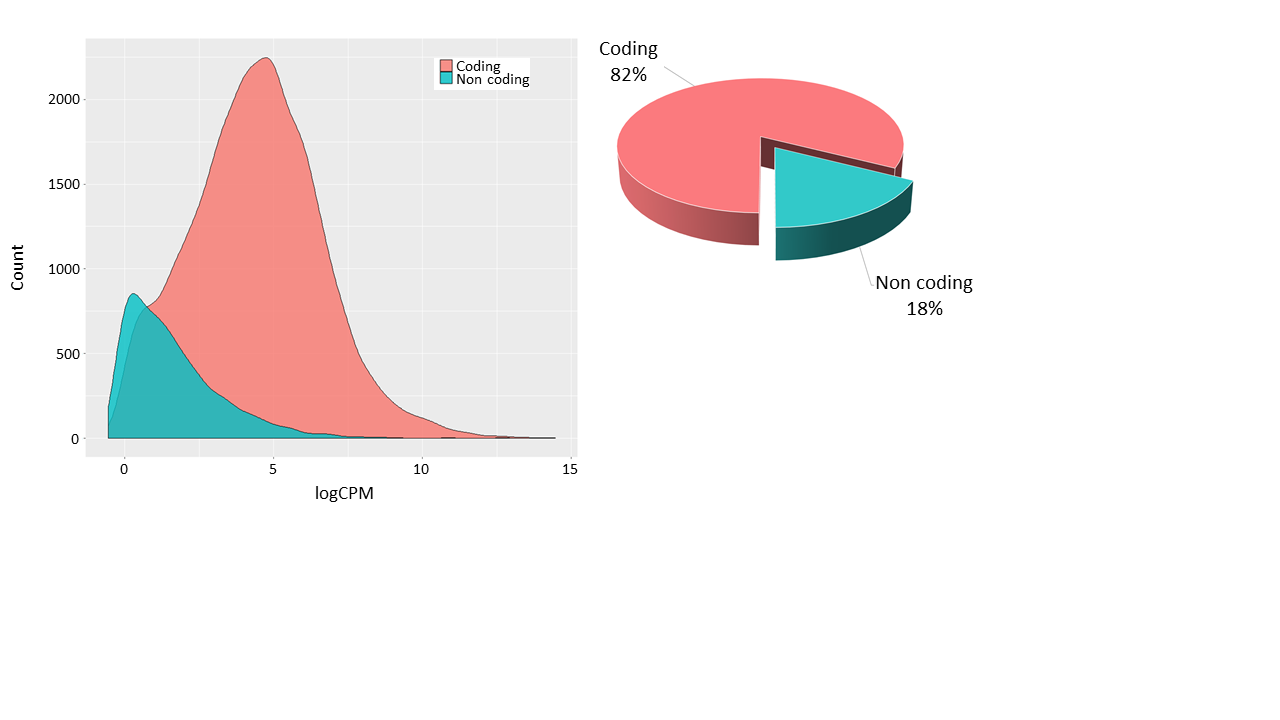

Supplement: FIGURE S3 — Distribution of gene expression. (A) Density distribution of the gene expression levels, grouped by coding (pink) and non-coding (light blue) expressed genes. The protein-coding genes show a higher average expression value than non-coding genes. (B) Pie chart of the percentage of coding (pink) and non-coding (light blue) expressed genes; more than 80% of expressed genes are protein-coding. [file Image_3.TIF]

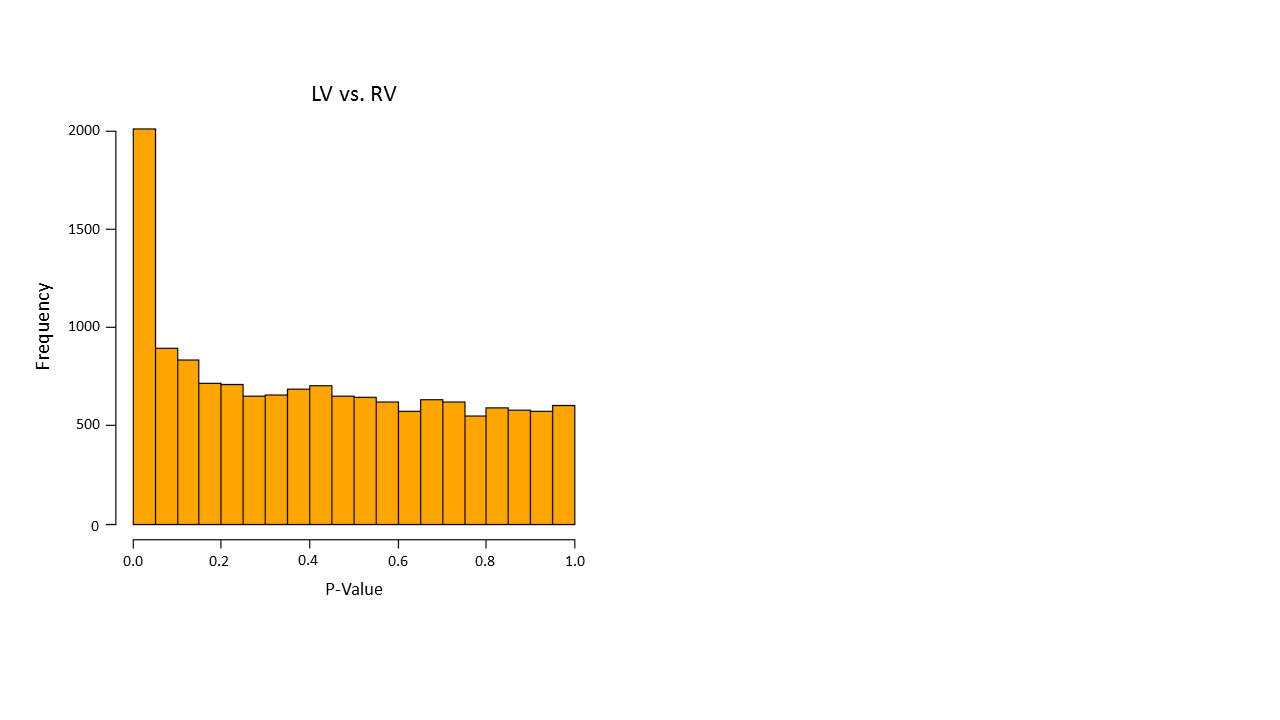

Supplement: FIGURE S4 — Histogram P-value. The histogram of P-values distribution for non-DE genes is ideally uniformly distributed across the unit interval, whereas the P-values for DE genes present a spike near zero. [file Image_4.TIF]
