# Supplementary material for: Human Cardiac Mesenchymal Stromal Cells From Right and Left Ventricles Display Differences in Number, Function, and Transcriptomic Profile
Source: Front Physiol. 2020 Jun 24;11:604. doi: 10.3389/fphys.2020.00604 (PMC7327120; doi:10.3389/fphys.2020.00604)
Supplement: TABLE S1 — Summary of the clinical features of the healthy controls from which LV and RV samples were obtained. [file Table_1.DOCX]

Supplementary Material

| **ID** | **Sex (M=male; F=female)** | **Age** | **Cause of death** | **Concomitant diseases** | **Drugs** | **Cardiovascular risk factors** |
| --- | --- | --- | --- | --- | --- | --- |
| **HC1** | M | 21 | Multiple trauma | / | / | / |
| **HC2** | M | 57 | Multiple trauma | Prostatic hypertrophy; coronary artery disease | Alpha blockers | Smoking |
| **HC3** | M | 57 | Cerebral hemorrhage | / | / | / |
| **HC4** | M | 44 | Cerebral hemorrhage | / | / | / |
| **HC5** | M | 18 | Multiple trauma | / | / | / |
| **HC6** | F | 50 | Drowning | Depression syndrome | / | / |
| **HC7** | F | 60 | Cerebral hemorrhage | / | / | / |
| **HC8** | F | 58 | Cerebral hemorrhage | Hypertension | / | Smoking |
| **HC9** | F | 50 | Respiratory failure | Idiopathic pulmonary fibrosis | Angiotensin Receptor Blockers | / |
| **HC10** | M | 18 | Multiple trauma | / | / | / |
| **HC11** | F | 58 | Cerebral hemorrhage | / | / | / |
| **HC12** | F | 18 | Multiple trauma | / | / | / |
| **HC13** | M | 20 | Multiple trauma | / | / | / |

**Table S1.** **Summary of the clinical features of the healthy controls from which LV and RV** **samples were obtained**

**Table S2.** **Primary antibodies**

| **Protein** | **Product code** | **Host** | **Company** | **Dilution** |
| --- | --- | --- | --- | --- |
| **CD29** | NCL-CD29 | Mouse IgG_1_ | Leica | IF 1:40 |
| **CD44** | ab119335 | Rat | Abcam | IF 1:200 |
| **CD105** | ab49679 | Mouse | Abcam | IF 1:100 |
| **CD14** | 641394 | Mouse IgG_2b_ | BD | FACS |
| **CD29** | 1P-219-T100 | Mouse IgG_1_ | EXBIO | FACS |
| **CD31** | 555445 | Mouse IgG_1_ | BD | FACS |
| **CD34** | 555822 | Mouse IgG_1_ | BD | FACS |
| **CD44** | 550989 | Mouse IgG_1_ | BD | FACS |
| **CD45** | 555485 | Mouse IgG_1_ | BD | FACS |
| **CD90** | 555596 | Mouse IgG_1_ | BD | FACS |
| **CD105** | 562408 | Mouse IgG_1_ | BD | FACS |
| **HLA-DR** | 347363 | Mouse IgG_2a_ | BD | FACS |
| **PPARγ** | sc-7273 | Mouse IgG_1_ | Santa Cruz | WB 1:60 |
| **FABP4** | ab93945 | Mouse IgG_1_ | Abcam | WB 1:1000 |
| **PLIN1** | BP5015 | Guinea Pig | Origene | WB 1:1000 |
| **COL1A1** | 84336 | Rabbit | Cell Signaling | WB 1:1000 |
| **GAPDH** | sc-25778 | Rabbit | Santa Cruz | WB 1:1000 |

**Table S3.** **Secondary antibodies**

| **Protein** | **Product code** | **Host** | **Company** | **Dilution** |
| --- | --- | --- | --- | --- |
| **Anti-mouse 594** | A-11032 | Goat | Life Technologies | IF 1:500 |
| **Anti-rat IgG 546** | A-11081 | Goat | Life Technologies | IF 1:200 |
| **Anti-mouse 488** | A-11001 | Goat | Life Technologies | IF 1:200 |
| **Anti-mouse HRP** | NA9310V | Sheep | GE healthcare | WB 1:1000 |
| **Anti-rabbit HRP** | NA9340V | Donkey | GE healthcare | WB 1:1000 |
| **Anti-guinea pig HRP** | ab6908 | Goat | Abcam | WB 1:4000 |

**Table S4.** **Primers**

| ***PPARγ*** | ACATAAAGTCCTTCCCGCTGACCA | AAACTGGCAGCCCTGAAAGATGC |
| --- | --- | --- |
| ***FABP4*** | TTCATACTGGGCCAGGAATTT | TCCATCCCATTTCTGCACAT |
| ***PLIN1*** | CATTGAGAAGGTGGTGGAGTA | CTTGGCCTTGGGAGACTT |
| ***GAPDH*** | CCACCCATGGCAAATTCC | TCGCTCCTGGAAGATGGTG |

**Table S5. Detailed FACS analysis of LV and RV C-MSC**

|  | **LV**  **n=6** | **RV**  **n=6** | p |
| --- | --- | --- | --- |
| **CD29** | 98.77±0.51 | 98.82±0.37 | ns |
| **CD105** | 96.90±1.03 | 98.10±0.70 | ns |
| **CD44** | 97.77±0.80 | 98.11±0.58 | ns |
| **CD90** | 18.11±10.36 | 21.39±9.83 | ns |
| **CD31** | 1.48±0.78 | 0.33±0.24 | ns |
| **CD34** | 0.26±0.19 | 0.33±0.27 | ns |
| **CD14** | 0.29±0.11 | 0.44±0.31 | ns |
| **CD45** | 0.10±0.04 | 0.20±0.13 | ns |
| **CD140a** | 3.48±2.79 | 13.44±10.08 | ns |
| **HLA-DR** | 0.20±0.07 | 0.47±0.37 | ns |

**Table S6. Summary of gene and protein expression during LV and RV C-MSC adipogenic differentiation for 72 hours and 1 week.** 2^-ΔΔ Ct ratio is reported for qRT-PCR analysis. The densitometric analysis for each protein normalized on GAPDH expression is shown for WB data.

|  |  | **LV**  **n=6** | **RV**  **n=6** | p |
| --- | --- | --- | --- | --- |
|  | ***PPARγ*** | 1.00±0.18 | 0.83±0.37 | ns |
| **72h** | ***FABP4*** | 1.00±0.50 | 0.56±0.36 | ns |
|  | ***PLIN1*** | 1.00±0.40 | 1.25±1.02 | ns |
|  | ***PPARγ*** | 1.19±0.35 | 1.82±1.11 | ns |
| **1w** | ***FABP4*** | 2.94±1.30 | 2.73±2.18 | ns |
|  | ***PLIN1*** | 0.97±0.46 | 2.18±1.98 | ns |
|  | **PPARγ** | 1.00±0.06 | 0.97±0.03 | ns |
| **72h** | **FABP4** | 1.00±0.18 | 0.98±0.23 | ns |
|  | **PLIN1** | 1.00±0.20 | 0.86±0.16 | ns |
|  | **PPARγ** | 0.90±0.08 | 0.91±0.09 | ns |
| **1w** | **FABP4** | 2.66±0.98 | 1.74±0.46 | ns |
|  | **PLIN1** | 1.24±0.16 | 2.28±0.87 | ns |

**Western Blot**

**qRT-PCR**
